# The accuracy of diagnostic coding for acute kidney injury in England – a single centre study

**DOI:** 10.1186/1471-2369-14-58

**Published:** 2013-03-13

**Authors:** Laurie A Tomlinson, Alex M Riding, Rupert A Payne, Gary A Abel, Charles R Tomson, Ian B Wilkinson, Martin O Roland, Afzal N Chaudhry

**Affiliations:** 1 Clinical Pharmacology Unit, Cambridge University Hospitals NHS Foundation Trust, Cambridge, UK; 2 Department of Nephrology, Cambridge University Hospitals NHS Foundation Trust, Cambridge, UK; 3 Cambridge Centre for Health Services Research, Institute of Public Health, Cambridge, UK; 4 The Richard Bright Renal Unit, North Bristol NHS Trust, Bristol, UK

**Keywords:** Acute kidney injury, Acute renal failure, ICD-10, Coding

## Abstract

**Background:**

Acute kidney injury (AKI) is an independent risk factor for mortality and is responsible for a significant burden of healthcare expenditure, so accurate measurement of its incidence is important. Administrative coding data has been used for assessing AKI incidence, and shows an increasing proportion of hospital bed days attributable to AKI. However, the accuracy of coding for AKI and changes in coding over time have not been studied in England.

**Methods:**

We studied a random sample of admissions from 2005 and 2010 where ICD-10 code N17 (acute renal failure) was recorded in the administrative coding data at one acute NHS Foundation Trust in England. Using the medical notes and computerised records we examined the demographic and clinical details of these admissions.

**Results:**

Against a 6.3% (95% CI 4.8-7.9%) increase in all non-elective admissions, we found a 64% increase in acute renal failure admissions (95% CI 41%-92%, p < 0.001) in 2010 compared to 2005. Median age was 78 years (IQR 72–87), 11-25% had a relevant pre-admission co-morbidity and 64% (55-73%) were taking drugs known to be associated with AKI. Over both years, 95% (91-99%) of cases examined met the Kidney Disease: Improving Global Outcomes criteria for AKI.

**Conclusions:**

Patients with hospital admissions where AKI has been coded are elderly with multiple co-morbidities. Our results demonstrate a high positive predictive value of coding data for a clinical diagnosis of AKI, with no suggestion of marked changes in coding of AKI between 2005 and 2010.

## Background

Acute kidney injury (AKI), previously called acute renal failure, is a sudden reduction in kidney function which can be due to a wide range of conditions including severe infection and hypovolaemia, or in response to certain drugs and toxins. Although AKI may result in complete recovery of kidney function, many patients require temporary dialysis or haemofiltration and prolonged hospital stay, and it is associated with a substantial mortality [1]. The net result is a significant burden on healthcare expenditure [2].

Despite the clinical importance of AKI, there are limited data to assess its incidence. Many large studies have used case definitions based on biochemical values and changes in urine output which are the gold-standard but have been complicated by changing definitions over time and may be logistically complex to analyse [3,4]. Other studies have used electronically recorded diagnostic codes such as the International Classification of Diseases (ICD), to quantify the burden of AKI [5,6]. In England, this data is available from Hospital Episode Statistics (HES), a database containing details of all admissions to National Health Service hospitals [7]. HES contains administrative details which have been electronically recorded for the vast majority of hospital admissions in England including diagnostic information, currently coded using the 10th edition of ICD (ICD-10). Such data is widely used for health services research, particularly where no biochemical information is available such as within primary care databases. Such research is important: based on coding data, the incidence and proportion of hospital bed days attributable to AKI has increased rapidly over the last 4 years [8] and these results have been used to argue for significant changes to health services and increased investment in care for AKI [9]. However, the apparent increase in hospital admissions due to AKI may be due to patients being wrongly coded, or to a change in clinical administrative practices, where patients are coded with AKI for a less severe kidney insult than previously. This could occur due to increased awareness of the condition, or result from ‘gaming’, since hospitals are remunerated according to patient codes. Therefore understanding the validity of such coding is vital but few studies have been conducted to examine the validity of diagnostic codes for AKI [10]. In particular, these have not been conducted in a United Kingdom setting and do not reflect changes over time. We therefore chose to examine in detail the clinical and biochemical characteristics of representative samples of patients who had an admission coded as AKI from two calendar years, 2005 and 2010 and to determine whether these patients did have AKI based on current criteria.

## Methods

Data obtained from HES for a hospital admission is comprised of one or more consecutive “episodes” of care (a period of care under a specific clinician). Each episode records a primary diagnosis (the main condition treated or investigated during this episode) and up to 19 additional secondary diagnoses.

We obtained a list of adult admissions to Addenbrooke’s Hospital in Cambridge, England during the full calendar years 2005 and 2010, where ICD-10 code N17 (acute renal failure) was listed in any diagnostic position in any admission episode. Since ICD-10 was introduced, the term AKI has largely replaced acute renal failure in clinical use but this has not yet been amended for coding purposes. Where multiple admissions for one individual occurred within the same year, only the first admission was included. We obtained a random sample of the admissions for both years and the same observer (AMR), an experienced clinician who was blinded to the study hypothesis, examined all relevant clinical records. Sample size was pragmatically chosen to enable a moderately precise estimate of patient characteristics, but without the power to detect differences between years. Patients who had been on dialysis or had a renal transplant prior to the relevant admission were excluded. The observer completed a predefined detailed summary of the demographic and clinical admission details, accessing additional computerised laboratory records where necessary.

We recorded the presence of a number of co-morbidities (diabetes, hypertension, cardiovascular disease and heart failure) based on a diagnosis made prior to admission and recorded in the hospital notes or general practitioner (GP) referral letter. Baseline estimated glomerular filtration rate (eGFR) was calculated from the lowest recorded value of creatinine in the three months prior to admission, or the last recorded value prior to this if no measurements were available during this period, using the 4-variable MDRD equation [11]. Since eGFR is not appropriate for assessment of renal function during episodes of AKI we quantified admission renal function using the first value of creatinine from a biochemistry sample analysed by the hospital pathology laboratory. All samples were measured within 24 hours of admission. Bicarbonate was the first recorded sample taken on the day of admission, whether taken from an arterial or venous blood gas sample. Systolic blood pressure was as recorded on the emergency department triage card or in the initial medical or nursing assessment. Recent diarrhoea or vomiting was recorded if a relevant history was given by the patient of either or both symptoms in the week preceding admission. We derived the pre-admission drug history from information recorded from the patient, GP letter or subsequently documented by a pharmacist in the medical notes after liaison with the GP practice. We evaluated drugs that are in common use in the community and known to be associated with AKI [12,13].

The observer compared the biochemical details of the entire admission to any of the Kidney Disease: Improving Global Outcomes (KDIGO) definitions of acute kidney injury (AKI) [14]. These were an increase in serum creatinine by ≥26.5 μmol/l within 48 hours, an increase in serum creatinine to ≥1.5 times baseline, an increase in serum creatinine to ≥354 μmol/L or commencement on renal replacement therapy, having met another definition of AKI. We also examined the criteria for urine output but biochemical and urine definitions were concordant and there were no situations where a case was defined by urine output alone. If the details of the admission were consistent with the KDIGO definition, the admission was defined as accurately coded.

This project was designated by Cambridge University Hospitals NHS Trust to be a service evaluation so individual informed patient consent was not required and ethical committee approval was not needed. Only one study team member (LAT) and the observer had access to identifiable patient information and this was kept confidential at all times. All other study members examined anonymised patient data only.

On inspection we found many continuous data items have had non-normal skewed distributions and so all such data are summarised as median (interquartile range, IQR). Dichotomous variables are presented as number and frequency with 95% confidence intervals (95% CI). Changes in admission rates were derived from Poisson regression with year as the only covariate.

## Results

### Numbers of admissions

There was a relatively small increase (6.3%, 95% confidence interval 4.8-7.9%) in non-elective admissions to Addenbrooke’s Hospital between 2005 and 2010, from 35,472 to 37,720. However, there was a disproportionate increase in AKI admissions (ICD-10 code N17 in any diagnostic position) from 261 to 429 (64%, 41-92%). 116 patients (44%, 38-50%) had the code N17 recorded in the primary diagnostic position, rather than a secondary diagnostic position, in 2005. Similar findings were observed in 2010 (44%, 39-48%). We examined 58 case notes from 2005 and 63 from 2010.

### Patient characteristics

Details of patients’ characteristics for each year are shown in Table 1. Across both years, the median age of patients was 78 years (IQR 72–87) and 48% (95% CI 39-57%) of patients were male. Rates of diabetes, hypertension, cardiovascular disease and heart failure were 30% (95% CI 22-38%), 38% (95% CI 29-47%), 31% (95% CI 22-39%) and 16% (95% CI 9-22%) respectively. Overall, 64% (95% CI 55-73%) of patients were on at least one drug that can increase the risk of AKI, and 22% (95% CI 15-30%) of patients had suffered from recent diarrhoea/vomiting.

**Table 1:** Summary of patient details

	**2005**		**2010**	
	**N (%)**	**95% CI**	**N (%)**	**95% CI**
**Number of records examined**	58 (100%)		63 (100%)	
**Died during admission**	30 (50%)	37-63%	29 (46%)	33-59%
**Male gender**	21 (36%)	23-49%	15 (24%)	13-35%
**Diabetic**	25 (43%)	30-56%	21 (33%)	21-45%
**Hypertensive**	18 (31%)	19-43%	19 (30%)	19-42%
**Previous IHD or CVD**	11 (19%)	9-29%	8 (13%)	4-21%
**Heart failure**	13 (22%)	11-33%	14 (22%)	12-33%
**Recent diarrhoea/vomiting**	11 (19%)	9-29%	9 (14%)	5-23%
**Admitted to ITU**	10 (17%)	7-27%	9 (14%)	5-23%
**Haemodialysis/haemofiltration**	29 (50%)	37-63%	29 (46%)	33-59%
**Pre-admission drug treatment**				
**ACE Inhibitor**	19 (35%)	22-48%	14 (23%)	12-34%
**ARB**	5 (9%)	1-17%	9 (15%)	6-24%
**Loop diuretic**	29 (55%)	37-63%	18 (29%)	17-40%
**Thiazide diuretic**	5(9%)	1-16%	3 (5%)	0-10%
**NSAID**	6 (11%)	2-19%	3 (5%)	0-11%
**Spironolactone**	8 (15%)	5-24%	6 (10%)	2-18%
	Median (IQR)		Median (IQR)	
**Age (years)**	79 (70–85)		83 (73–88)	
**Biochemical variables**				
**Baseline eGFR (ml/min/1.73m**^**2**^**)**	49 (29–62)		45 (33–65)	
**Baseline creatinine (μmol/L)**	120 (90–170)		120 (90–155)	
**Admission creatinine (μmol/L)**	265 (178–405)		252 (191–435)	
**Admission potassium (mmol/L)**	5.0 (4.3-6.0)		4.5 (4.1-5.9)	
**Admission bicarbonate (mmol/L)**	17.2 (12.9-21.1)		21.5 (16.8-26.1)	
**Admission systolic BP (mmHg)**	122 (105–143)		127 (111–145)	

Count data are expressed as % (N), with 95% CI. Continuous data are expressed as median (IQR). *IHD* ischaemic heart disease; *CVD* cerebrovascular disease; *ITU* Intensive therapy unit; *eGFR* estimated glomerular filtration rate; *ACE* Angiotensin Converting Enzyme; *BP* blood pressure; A*RB* Angiotensin Receptor Blocker; *NSAID* Non-steroidal anti-inflammatory drug.

Median admission systolic pressure was 124 mmHg (IQR 107–143) across both years. Baseline creatinine was available in 52 records (88%) for 2005 and 58 records (94%) for 2010. Median baseline creatinine was 120μmol/L (IQR 90–160), with corresponding median baseline eGFR of 47ml/min/1.73m^2^ (IQR 32–64). Admission creatinine was only missing from one record in each of 2005 and 2010, median 257μmol/L (IQR 188–420) across both years. Bicarbonate was only available for 24 records (41%) for 2005, but 55 records (87%) for 2010, median 21mmol/L (IQR 16–25) across both years.

Overall, 17% (95% CI 10-23%) of patients required admission to the intensive care unit, 16% (95% CI 9-22%) received haemodialysis or haemofiltration, and 49% (95% CI 40-58%) died during admission.

### Accuracy of coding

In total, over both years, the percentage of cases meeting the KDIGO criteria for AKI (positive predictive value) was 95% (95% CI 91-99%). Rates were similar for 2005 (95%, 95% CI 89-100%) and 2010 (94%, 95% CI 88-100%). The positive predictive value for patients who survived to discharge across both years was 95% (95% CI 90-100%).

## Discussion

The main finding of this study is that the accuracy of coding of AKI is very good, with 95% of cases meeting the KDIGO definition. The high positive predictive value for diagnosis is consistent with previous American studies which found a value of 94% for the ICD-9 code for acute renal failure requiring dialysis [15] and 99% for ‘renal failure’ [16].

Our results also show that there has been a 64% increase in admissions with acute renal failure coded as either a primary or secondary diagnosis in 2010 compared to 2005. No English data are available for comparison but our findings are consistent with an increase in the percentage of bed days attributable to AKI over that time period and increased admissions described in America [8,17]. It is unclear if our results reflect a true increase in incidence or are due to greater recognition of AKI, with a higher proportion of true cases being identified by coding in 2010. The study was not designed or powered to detect differences in patient characteristics between the two years examined. Indeed, we found no statistical evidence (results not shown) of substantial differences in patient characteristics, biochemical parameters, clinical outcomes or pre-admission drug use patterns between 2005 and 2010. However, it is worth noting that biochemical values and complication rates may be suggestive of lower disease severity in 2010 than in 2005; a larger sample size would be required to confirm this. Alternately, improved early diagnosis and management of patients with AKI may contribute to these differences [18].

Strengths of this study are the use of a representative sample of cases admitted to a large teaching hospital, reviewed in detail in a blinded manner by an experienced clinician, and detailed characterisation of the patients. There are important limitations however, including its retrospective nature, limited size and restriction to the coding practice of a single centre. The inpatient mortality of this sample is higher than that seen nationally [5], and the results may therefore not be generalizable to a less seriously ill population. This may in part reflect ease of obtaining notes since case records of deceased and living patients are kept separately. The proportion of deceased patients could bias our results if patients were more likely to be wrongly coded depending on whether they lived or died. However, the positive predictive value of a code of AKI for the KDIGO definition among patients who survived to discharge across both years was the same as seen in the whole cohort, suggesting that substantial bias is unlikely to have occurred.

Data were not complete for every case examined although overall the amount of missing data was small. Although the observer was blinded to the hypothesis, she was not blinded to the attributed code which may have resulted in bias to confirm a diagnosis of AKI. We only examined the specificity of the N17 code (acute renal failure), and not other codes to which cases of AKI may have been attributed (e.g. N19; unspecified kidney failure). For a small proportion of the cases, patients did not have AKI on admission but developed it subsequently during their hospital stay. Therefore, while we considered whether the diagnostic criteria for AKI were met at any time during the admission, the biochemical changes may not be reflected in the admission characteristics presented in this paper. We did not investigate the incidence of AKI in patients who were not coded with N17 and therefore cannot comment on the specificity or sensitivity of the code for a diagnosis of AKI, or the characteristics of cases of AKI that were not coded.

Acute kidney injury is often preventable, associated with high healthcare costs and is an important risk factor for mortality [4]. Therefore, clear understanding of the epidemiology of AKI is very important. The largest studies of AKI incidence and outcomes have been based on changes in serial creatinine measurements using the ‘Risk, Injury, Failure, Loss, End-Stage Renal Disease’ (RIFLE) and Acute Kidney Injury Network (AKIN) criteria [19,20]. While biochemical and clinical definitions are crucial for defining cases to examine AKI epidemiology, they are associated with a number of problems. These include the difficulty of defining baseline creatinine, difficulty in detecting additional cases defined only by a reduction in urine output and are subject to a number of biases such as more frequent blood testing in unwell patients. In addition, these definitions cannot be used in situations where limited biochemical data is available; for example, pharmacoepidemiological studies in primary care outpatient databases. Therefore the use of administrative coding data to examine AKI epidemiology may be crucial in some situations. However, studies are dependent on the validity of the coding and changes in coding practice over time, and may underestimate incidence if only more severe cases are coded [21].

## Conclusions

In a single large teaching hospital there was a 64% increase in hospital admissions coded with acute renal failure as the primary or secondary diagnosis in 2010 compared to 2005. For both years, the patients were elderly with a high level of co-morbidities and use of drugs known to be associated with AKI. A detailed analysis of a representative sample of these admissions found that 95% met international diagnostic criteria for AKI. Our results demonstrate a high positive predictive value of coding data for a clinical diagnosis of AKI, with no suggestion (accepting the limitations of the study) of marked changes in coding of AKI between 2005 and 2010.

## Competing interests

The authors declare they have no competing interests.

## Authors’ contributions

LAT conceived of the study, participated in its design and coordination, analysed and interpreted the data and wrote the initial draft of the manuscript. AMR carried out the analysis of patient notes and helped to draft the manuscript. GAA and RAP contributed to study design, statistical analysis and helped to draft the manuscript. ANC, CRT, IBW and MOR all contributed to study design and conception and helped draft the manuscript, revising it critically for important intellectual content. All authors read and approved the final manuscript.

## Funding

ANC is supported by the Cambridge Biomedical Research Institute and IBW by the British Heart Foundation.

## Pre-publication history

The pre-publication history for this paper can be accessed here:

http://www.biomedcentral.com/1471-2369/14/58/prepub
